# From NAFLD to MAFLD: Aligning Translational In Vitro Research to Clinical Insights

**DOI:** 10.3390/biomedicines10010161

**Published:** 2022-01-12

**Authors:** Alexandra Gatzios, Matthias Rombaut, Karolien Buyl, Joery De Kock, Robim M. Rodrigues, Vera Rogiers, Tamara Vanhaecke, Joost Boeckmans

**Affiliations:** Department of In Vitro Toxicology and Dermato-Cosmetology, Faculty of Medicine and Pharmacy, Vrije Universiteit Brussel, Laarbeeklaan 103, 1090 Brussels, Belgium; Matthias.Rombaut@vub.be (M.R.); Karolien.Buyl@vub.be (K.B.); Joery.De.Kock@vub.be (J.D.K.); Robim.Marcelino.Rodrigues@vub.be (R.M.R.); Vera.Rogiers@vub.be (V.R.); Tamara.Vanhaecke@vub.be (T.V.)

**Keywords:** NAFLD, MAFLD, NASH, liver, drug development, pharmacology, in vitro

## Abstract

Although most same-stage non-alcoholic fatty liver disease (NAFLD) patients exhibit similar histologic sequelae, the underlying mechanisms appear to be highly heterogeneous. Therefore, it was recently proposed to redefine NAFLD to metabolic dysfunction-associated fatty liver disease (MAFLD) in which other known causes of liver disease such as alcohol consumption or viral hepatitis do not need to be excluded. Revised nomenclature envisions speeding up and facilitating anti-MAFLD drug development by means of patient stratification whereby each subgroup would benefit from distinct pharmacological interventions. As human-based in vitro research fulfils an irrefutable step in drug development, action should be taken as well in this stadium of the translational path. Indeed, most established in vitro NAFLD models rely on short-term exposure to fatty acids and use lipid accumulation as a phenotypic benchmark. This general approach to a seemingly ambiguous disease such as NAFLD therefore no longer seems applicable. Human-based in vitro models that accurately reflect distinct disease subgroups of MAFLD should thus be adopted in early preclinical disease modeling and drug testing. In this review article, we outline considerations for setting up translational in vitro experiments in the MAFLD era and allude to potential strategies to implement MAFLD heterogeneity into an in vitro setting so as to better align early drug development with future clinical trial designs.

## 1. Introduction

The prevalence of non-alcoholic fatty liver disease (NAFLD) has risen over past decades, closely paralleling the obesity pandemic, making it the number one chronic liver disease today [1]. NAFLD is a slowly progressing disease, beginning with isolated liver steatosis that evolves in a subset of patients to non-alcoholic steatohepatitis (NASH), liver fibrosis, cirrhosis and hepatocellular carcinoma (HCC) [2]. NAFLD is defined as the presence of hepatic steatosis in at least 5% of the liver parenchyma, in the absence of other hepatic pathologies, such as viral hepatitis and without significant alcohol consumption or intake of steatogenic drugs (e.g., valproic acid, tamoxifen) [3].

Major risk factors for developing NAFLD are obesity and type II diabetes mellitus (T2DM), which is why NAFLD is often considered as the hepatic manifestation of the metabolic syndrome. NAFLD patients typically exhibit serum lipid dysregulation characterized by increased levels of low-density lipoprotein (LDL) particles and a reduced amount of high-density lipoproteins (HDL) [4,5]. Unsurprisingly, cardiovascular disease is one of the most common causes of death in NAFLD patients. Because of the unmet need for effective anti-NAFLD therapeutics, hundreds of clinical studies have been carried out in the past decade, however, without satisfying results [6]. Clinical studies report drug-response rates ranging from 20–40% with placebo effects accounting for 10 to 20% [7,8], suggesting the presence of certain effect modifiers.

Weight loss through lifestyle modification, achieved through close follow-up by dieticians and physical activity, might be a cost-effective and efficient approach for treatment of NAFLD [9]. Consequently, one solution to avoid noise in clinical studies could be standardization of diet and exercise during anti-NAFLD treatment [10]. Yet, one may not forget that certain NAFLD/NASH patients are lean or exhibit other non-obesity related causes of NAFLD for which weight loss is not an option. Further, the desired lifestyle modifications and weight loss are often hardly attainable outside well-controlled settings. In addition, it has become clear that NAFLD should be better understood and that its pathogenesis is likely much more heterogenic than previously assumed [11]. In this context, the NAFLD nomenclature was recently revised to ‘metabolic dysfunction-associated fatty liver disease (MAFLD)’, in order to avoid diagnosis based on exclusion of other pathologies and to better mirror its pathogenesis [7].

**Figure 1:**
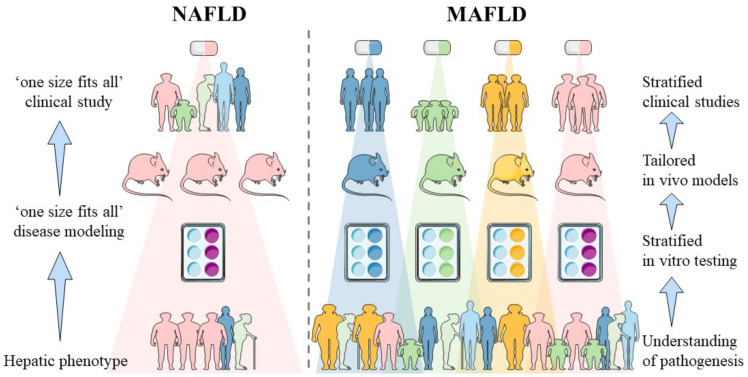
Impact of MAFLD nomenclature on different stages of drug development. Until now, patient inclusion for anti-NAFLD clinical studies was based on hepatic phenotype in the absence of significant alcohol intake and concurring pathologies. Anti-MAFLD trials will be undertaken based on pathogenic determinants that will likely require distinct pharmacological interventions. Introduction of the MAFLD terminology requires deeper understanding of the pathogenesis of fatty liver disease and hence necessitates the development of human-based in vitro models able to represent the distinct disease subsets. Disease subsets of MAFLD are indicated by the blue, green, yellow and pink colors. Corresponding in vitro tests, in vivo models, stratification in clinical studies and distinct pharmacological interventions are visualized by the same color for each disease subset. [Abbreviations: MAFLD: Metabolic-dysfunction associated fatty liver disease, NAFLD: non-alcoholic fatty liver disease].

The diagnosis of MAFLD is made based on the presence of hepatic steatosis with at least one of three of the following features: (i) obesity/overweight, (ii) diabetes mellitus and/or (iii) evidence of metabolic dysregulation [7,12]. The origin of MAFLD is, however, unspecified and highly heterogeneous due to multiple coexisting disease-modifiers including sex, age, diet, alcohol consumption, concurring liver pathology, genetic background and metabolic pressure. Most patients suffering from NAFLD also meet the criteria for MAFLD. A recent cross-sectional study revealed that the NAFLD prevalence was 37.1% (95% CI, 34.0–40.4), whereas the MAFLD prevalence was slightly higher at 39.1% (95% CI, 36.3–42.1) in the general population of the United States of America [13]. As suggested by Eslam et al., novel clinical trial design will consist of stratified patient groups based on MAFLD etiology rather than inclusion based on histology and scoring systems [7]. Therefore, patient heterogeneity should also be adopted in preclinical disease modeling and drug screening studies. The impact of the change in terminology on different stages of drug development is visualized in Figure 1.

In the past decades, a variety of human-based hepatic in vitro models have been developed and applied in preclinical anti-NAFLD drug development. Nevertheless, these established models, of which a non-limitative list is shown in Table 1, have important drawbacks when considering the change from NAFLD to MAFLD nomenclature. Indeed, most traditional NAFLD in vitro models are based on short-term exposure to fatty acids after which accumulation of lipids is taken as a phenotypic benchmark of NAFLD [2]. Although the former approach could reflect obesity-related and diet-induced MAFLD, other etiologies of MAFLD should be modeled separately in vitro to tailor drug development to the underlying cause of the disease subtype.

Next to the cell source and in vitro disease triggers, each cell culture system also has its specific strengths and limitations when it comes to in vitro MAFLD modelling. These characteristics should be taken into account when selecting the most suitable in vitro model for each specific application [26]. Table 2 summarizes the most important strengths and drawbacks of currently available hepatic in vitro cell culture systems.

In this review article, we allude to the implications of the change in NAFLD nomenclature for translational in vitro research. Further, we outline strategies for integrating essential aspects of MAFLD into an in vitro setting in order to better align preclinical experiments to future clinical studies.

Most research has been published in the context of the established NAFLD terminology. Although MAFLD mostly covers patients that meet the criteria for NAFLD, key differences exist in their definition and diagnostic criteria and, therefore, these terms should not be used interchangeably. Throughout this review article, we adopted the terminology used by the authors of each cited publication and only moved to the MAFLD terminology when discussing future perspectives and suggestions for applying this new nomenclature to in vitro preclinical research.

## 2. Sources of Heterogeneity in MAFLD and Their In Vitro Implementation

### 2.1. Sex and Hormonal Status

NAFLD occurs more often in males, in whom higher liver pyruvate kinase levels positively correlate with NAFLD severity [33,34]. A recent transcriptomic study also points to the fact that NASH is a sexual dimorphic disorder that might lead to different drug responses between men and women [35]. Remarkably, the phase II trial in which the dual C-C motif chemokine receptor (CCR) 2/5 antagonist cenicriviroc was evaluated showed a positive effect in men regarding fibrosis regression after one year, but not in women, although this was not necessarily associated with response to cenicriviroc [36]. Based on these findings, it can be presumed that anti-NASH treatment likely requires a sex-specific approach and thus the need for both female- and male-based preclinical models. Moreover, as gender-affirming hormone therapy has also been shown to lead to altered metabolic profiles [37], but little is known about the impact of such therapies on MAFLD development, in vitro models might prove useful to gain further insights on this topic. As sex hormones such as estrogens and androgens are known to influence lipid metabolism [38], their inclusion in in vitro MAFLD models could be a first step towards sex-specific MAFLD models. Hence, it is also important that the sex of the donor from whom cells are obtained for in vitro modeling is known. Although the source of primary human hepatocytes is most often well-documented, study results are generally not placed into context and interpreted in a unisex manner. Furthermore, commonly used human hepatic cell lines originate from sources from different sexes: HepG2 cells were isolated in 1975 from a hepatocellular blastoma from a 15-year old boy [39], whereas HepaRG cells were isolated from a HCC of a female patient [40]. Since a large part of the scientific literature relies on data obtained using these cell lines [2], one might question whether existing data regarding NAFLD should be reinterpreted in a sex-specific context.

### 2.2. Pediatric/Juvenile MAFLD

NAFLD prevalence positively correlates with aging [34,41,42]; however, pediatric NAFLD seems a distinct entity with a more severe phenotype [41]. Indeed, NASH-related cirrhosis has been reported already in children of eight years old [42]. Pediatric NAFLD patients exhibit greater periportal inflammation, which is associated with hyperuricemia, while adult NAFLD mainly manifests in perivenous zones [43]. Pediatric periportal steatosis associates with advanced fibrosis, while perivenous steatosis correlates with steatohepatitis. Of these subgroups, children suffering from periportal steatosis are significantly younger than the ones suffering from perivenous steatosis (ten vs. fourteen years old, respectively) [44].

Reportedly, 8% of children from the general population and up to 34% of obese children suffer from NAFLD globally [45,46,47]. Between 1990 and 2017 the annual increase in juvenile NAFLD prevalence was 1.35% and although this increasing trend is observed worldwide, it is most pronounced in regions with a high-middle socio-demographic index [47]. Considering the increasing prevalence of NAFLD among children, in vitro models could provide insights in the underlying mechanisms that drive pediatric MAFLD and aid drug development for this specific subgroup. One way to achieve this is to use iPSC generated from cells collected from children in a non-invasive way (e.g.*,* urine cells) [48,49].

### 2.3. Fructose Consumption

In the past decade, excessive dietary fructose consumption has been identified as a driver of NAFLD development and progression. Fructose-related liver steatosis and NASH are independent from metabolic syndrome or obesity, which makes it a silent and widely spread risk factor [50,51,52]. Fructose intake is, however, associated with the development of features of the metabolic syndrome, such as elevated serum triglycerides and insulin resistance [53,54]. Fructose is metabolized in vivo by the liver and therefore it can be easily introduced to current in vitro models by direct (sequential) exposure of the hepatic cells through the culture medium [55]. Indeed, human co-cultures consisting of primary hepatocytes, stellate cells, hepatic sinusoidal endothelial cells and biliary epithelial cells have been exposed to glucose and fructose and shown to exhibit increased expression of lipogenic and fibrogenic genes. This can be reversed by inhibiting ketohexokinase, which intervenes in the enzymatic phosphorylation of fructose to fructose-1-phosphate [56]. Nonetheless, not all NASH-promoting effects of fructose are mediated by the aforementioned cell types. Fructose also promotes endotoxemia and activation of MYD88 innate immune signal transduction adaptor (MYD88)-mediated inflammatory signaling in liver myeloid cells resulting in the generation of tumor necrosis factor alpha (TNF-α). TNF-α subsequently stimulates caspase 2 and endoplasmic reticulum (ER)-stress, which in turn triggers the lipogenic transcription factor sterol regulatory element-binding protein-1c (SREBP-1c) to initiate de novo lipogenesis. Thus, modeling mechanisms of fructose-mediated MAFLD requires more complex disease models that include different cell types, or well-defined culture media that mimic the presence of non-parenchymal cells, likely consisting of fructose, endotoxins and TNF-α, of which the latter seems the most important trigger of lipogenesis [17].

### 2.4. Genetic Predisposition and Ethnicity

Important racial and ethnic disparities exist in NAFLD prevalence and risk for progression to end-stage liver disease [57]. The prevalence of NAFLD is the highest among the Hispanic population, the lowest in the black population and intermediate in the white population. Similarly, in comparison to white people, the risk for progression to NASH is the highest in Hispanic people (relative risk, 1.09; 95% confidence interval (CI), 0.98–1.21) and the lowest in black individuals (relative risk, 0.72; 95% CI, 0.60–0.87) in comparison to the white population, although without a difference in fibrosis occurrence [58]. The carriage of certain single nucleotide polymorphisms (SNPs) has also been linked to NAFLD development and disease severity. Hitherto, the carriage of *patatin-like phospholipase domain-containing protein 3* (*PNPLA3*) rs738409 appears to be the strongest genetic disease modifier [59]. Other SNPs that have been linked to NASH development and progression, include *transmembrane 6 superfamily member 2* (*TM6SF2*) rs58542926, involved in very low-density lipoprotein (VLDL) lipidation and secretion, and *membrane bound O-acyltransferase domain containing 7* (*MBOAT7*) rs641738, involved in phospholipid acyl-chain remodeling [60,61,62,63]. Of note, *PNPLA3* rs738409 is highly prevalent among the Hispanic population [58]. Previous in vitro and clinical research showed that PNPLA3 rs738409 has desaturating activity towards liver triglycerides, because it impairs hydrolysis/transacylation of poly-unsaturated fatty acids from diacylglycerols for synthesis of phosphatidylcholine [64]. In addition, homozygous carriage of *PNPLA3* rs738409 promotes the pro-fibrogenic properties of hepatic stellate cells [65]. Therefore, cellular systems that carry this specific polymorphism are of particular interest to model MAFLD and could enable personalized testing of anti-NASH pharmaceuticals. Recent in vitro research, using induced pluripotent stem cell (iPSC)-derived hepatocyte-like cells with knocked in *PNPLA3* rs738409, showed that this polymorphism induces a loss of function of PNPLA3 and thus impairs lipid metabolism, thereby predisposing these cells to steatosis. Furthermore, due to downregulation of enzymes involved in xenobiotic metabolism and excretion, *PNPLA3* rs738409 also increases sensitivity to hepatotoxins. This could explain how subclinical but sustained hepatic damage, attributed to environmental hepatotoxins, may result in disease progression towards NASH in patients carrying *PNPLA3* rs738409 [66]. For these reasons, cellular models for either dissecting the MAFLD pathogenesis or for testing novel therapeutics should be genetically characterized. As the ultimate example of in vitro translation of patient heterogeneity, it was demonstrated that iPSC-derived hepatocyte-like cells from four different donors, overloaded with oleic acid, also showed different lipid metabolism associated gene expression profiles related to the steatosis phenotype of the donor [20].

### 2.5. Epigenetics

Epigenetic alterations of gene expression, including DNA methylation, histone modifications and non-coding microRNA (miR) expression, are involved in underlying disease mechanisms of NASH and other metabolic diseases [67]. Liver miR-122 expression is 10-fold lower in NASH patients in comparison to simple steatosis, while in vitro its overexpression in transfected Huh7 cells results in higher alanine aminotransferase activity [68]. Various other miRs have also been associated with NASH, such as miR-223, which is elevated in NASH liver biopsy samples [69].

NASH patients exhibit significantly elevated levels of liver mitochondrial DNA methylation [70] and the latter is also involved in transdifferentiation of hepatic stellate cells to myofibroblasts, hence accompanying liver fibrosis [71]. Moreover, DNA methylation profiles could serve as a prognostic tool for NAFLD in the prevention of disease progression towards HCC [72]. *Tubulin*, *beta 2B class IIB* (*Tubb2b*) was found to be hypomethylated and overexpressed in a murine model investigating NASH-associated liver carcinogenesis. Markedly, *Tubb2b* was increasingly demethylated in the stages of NAFLD, NASH-fibrosis and HCC and this inversely correlated with *Tubb2b* gene expression at all of these stages. This phenomenon was also more present in HepG2 cells when compared to HepaRG cells, the latter being considered as better-differentiated hepatic cells. When culturing HepaRG cells in the presence of oleic acid, methylation of the *Tubb2b* promotor decreased along with increased expression of the gene. Overall, these data suggest that *Tubb2b* overexpression, due to increased demethylation, may be involved in the NAFLD pathogenesis and development of HCC [73].

Juvenile NAFLD probably already starts with epigenetic alterations and possibly other maternal-driven exposures in utero [7,74]. Human data are evidently scarce. Nevertheless, it was demonstrated that stillborn babies from mothers suffering from diabetes (type I, II and gestational) often exhibit hepatic steatosis [75]. Since epigenetic alterations can be passed to next generations or lost, one should keep track of the origin of stem cells, cell lines and primary cells used in both basic and translational MAFLD research [76].

### 2.6. Obesity and Body Fat Distribution

Obesity is one of the most common and well described risk factors for NAFLD [77]. Indeed, among adult NAFLD patients, the prevalence of obesity is estimated at 51.34% (95% CI, 41.3–61.20%) and the global increase in NAFLD prevalence is consistent with the alarming rise of obesity prevalence worldwide [78]. However, a subgroup of obese patients can be categorized as metabolically healthy and exhibit a significantly decreased risk of developing comorbidities [79,80]. Contrarily, up to 30% of adult normal weight individuals are metabolically obese and display increased cardiometabolic risk [81,82]. Therefore, it seems it is not the total amount of body fat but rather the distribution thereof that is an important disease driver. Indeed, visceral adipose tissue (VAT) area is associated with increased risk to develop NAFLD. To the contrary, larger areas of subcutaneous adipose tissue were found to associate with NAFLD regression in normal weight individuals [83]. Further, VAT area directly relates to hepatic inflammation and fibrosis development in NAFLD patients [84,85]. VAT is a hormonally active tissue that releases a spectrum of mediators that are involved in NAFLD such as adiponectin, interleukin 6 (IL-6) and TNF-α. Serum level of adiponectin, an anti-inflammatory and anti-lipogenic adipokine, is negatively correlated with VAT area [86] and was also found to be decreased in NASH patients [87]. In patients with metabolically unhealthy obesity, increased concentrations of IL-6 were found in VAT, suggesting increased pro-inflammatory activity of VAT [88,89]. Serum concentrations of IL-6 were also determined to significantly relate to VAT area [90]. Furthermore, IL-6 content in adipose tissue of metabolically unhealthy obese patients was found to be over one hundred-fold higher when compared to the liver content, suggesting that adipose tissue is the key source of IL-6 during metabolic dysregulation [88]. The degree of hepatitis and fibrosis positively correlates with both circulating and liver IL-6 concentrations [91]. From these data, it is clear that adipose tissue, and in particular VAT, is an important disease driver and should be encompassed in MAFLD in vitro models. A basic approach to recapitulate the influence of VAT-secreted mediators would be to expose human cell cultures to physiologically relevant concentrations of adiponectin, IL-6 and TNF-α in vitro. A recently proposed microfluidic device consisting of primary human hepatocytes and adipocytes was able to demonstrate indirect effects of adipose tissue on hepatocytes and seems promising for use in preclinical settings [92].

### 2.7. Lean MAFLD

Approximately 30% of normal weight individuals are metabolically unhealthy, which is also known as metabolic obesity. These persons exhibit a higher risk to develop metabolic diseases such as MAFLD [81,82]. In lean individuals worldwide, the NAFLD prevalence is 9.7% (95% CI: 7.7–11.8%), which is, however, in absolute proportions a significant number of NAFLD patients. Middle-aged patients in Asian countries show the highest prevalence of lean NAFLD [93]. Lean NAFLD patients often suffer from metabolic dysfunction and visceral adiposity [94], but also exhibit altered gut microbiota, higher secondary bile acid and FGF19 levels along with lower 7-alpha-hydroxy-4-cholesten-3-one compared to healthy persons and patients suffering from non-lean NAFLD [95]. Interestingly, when compared to obese NAFLD patients, the prevalence of the SNP *TM6SF2* rs58542926 seems to be higher in individuals suffering from lean NAFLD [95,96]. From these data, it is clear that lean MAFLD should be considered as a distinct entity with more than one metabolic determinant. Importantly, lean NAFLD in white individuals may progress to end-stage liver disease without associated *PNPLA3* rs738409 variant or progression to obesity, along with the full spectrum of metabolic comorbidities [97]. The true determinants of lean MAFLD are, however, still unknown. Mechanistic and translational in vitro studies regarding genetic determinants and drug response in this patient group therefore seem of high importance.

### 2.8. Microbiota

In the past decade, the gut-liver axis gained much attention as a key player in a variety of diseases. *Proteobacteria*, *Enterobacteria*, *Escherichia* [98] and *Bacteroides* [99] have been found to be more abundant in the stool of adult NASH patients than in healthy individuals. In stool samples of children with NAFLD, *Gammaproteobacteria* were found to be increased when compared to both obese and non-obese children without NAFLD [100]. Of note, NASH patients were found to exhibit increased serum levels of endogenous ethanol. Indeed, alcohol-producing microbiota such as *Escherichia* are more abundant in the stool of NASH patients [98]. Another study found that in a cohort of Chinese NAFLD patients, up to 60% of the patients were associated with high-alcohol-producing *Klebsiella pneumoniae* [101]. These findings suggest a role for these microbiota in the pathogenesis of NAFLD. Translocation of bacterial components to the liver can initiate and push MAFLD development. The best-known factor implicated in gut-related NASH is lipopolysaccharide (LPS) derived from Gram-negative bacteria [102]. LPS can induce hepatic inflammation by interacting with Toll-like receptors (TLRs) on both hepatocytes and hepatic stellate cells. For example, primary human hepatocytes challenged with LPS in vitro exhibited upregulation of IL-1β and IL-6 [103]. Additionally, upon exposure to LPS in vitro*,* LX-2 stellate cells displayed upregulation of *IL-1β*, *IL-6*, *TGF-β* and *TNF-α*, highlighting the proinflammatory effects of LPS [104]. Although in vitro direct exposure of cell systems to LPS has been demonstrated to be a practical approach [105], a more comprehensive way of working could be investigated. Indeed, one of the possibilities to more accurately model dysbiosis-driven MAFLD could be to expose human cell cultures to bacterial metabolites that are found at higher levels in the plasma of MAFLD patients, such as trimethylamine N-oxide, glycocholic acid and deoxycholic acid [106].

### 2.9. Zonation

The liver exhibits metabolic zonation [107]. Hepatocytes located close to the portal triad (zone 1) are more involved in gluconeogenesis and fatty acid β-oxidation, while hepatocytes in the pericentral region (zone 3) are rather involved in glycolysis and lipogenesis [108]. As such, steatosis generally occurs in the pericentral regions where the oxygen concentration is relatively lower [109]. However, in certain pediatric NAFLD patients, steatosis occurs in the periportal region and can be azonal as well [44]. Although lipid zonation seems lost in NASH [110], future research should clarify whether certain MAFLD phenotypes are of ‘pediatric’ or ‘adult’ nature and how oxygen tension and nutrient supply could modify lipogenesis in certain cell types. Furthermore, cells are commonly cultivated under 20–21% oxygen, while the partial oxygen tension in the liver ranges from 10–12% (periportal) to 3–5% (pericentral) [111]. This raises the question whether standard cultivation techniques for hepatic cells, whether or not for MAFLD research, should be revised [112]. As microfluidic devices support inclusion of zonation by limiting the flow rate, hereby altering oxygen and nutrient supply, they could provide valuable insights in this area of MAFLD research.

### 2.10. Disease Progression and Regression

**Figure 2:**
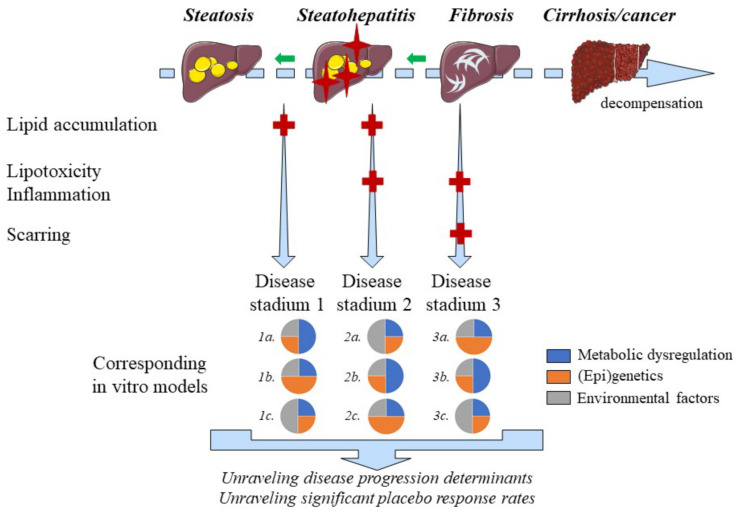
Positioning of MAFLD disease models. MAFLD is a naturally progressing and regressing disease of which different stadia can be accurately modeled in vitro. The main pathogenic drivers, however, differ among patients suffering from MAFLD, which requires multiple disease models for different MAFLD stadia. Steatosis is indicated by yellow ovals whereas red crosses on the liver figures indicate additional inflammation. Light blue fibers indicate hepatic accumulation of extracellular matrix components. The key factors involved in the different stages of MAFLD, mentioned on the left side of the figure, are indicated by corresponding red ‘+’ symbols. Regression of the disease is represented by the green arrows in the decompensation timeline.

MAFLD is a naturally progressing and regressing disease, which may partly explain high placebo response rates in clinical studies. In vitro models are well-positioned to study the MAFLD disease course, which seems key for unraveling the determinants of disease progression, regression and hence high placebo response rates (Figure 2) [2]. Hepatic steatosis is considered to precede inflammation across the spectrum of MAFLD. Yet, inflammation may also precede lipid accumulation [88], which is in conflict with traditional presumptions. Using iPSC-derived hepatic cells it was demonstrated that thapsigargin-mediated induction of ER-stress is an important driver of lipogenesis [113] and a pathway that should be considered when modeling steatosis-preceding inflammation. Stressed hepatocytes may accumulate lipids, which can easily be modelled in vitro using co-culture systems with macrophages and/or stellate cells [16]. Yet, increasing complexity, which is typical in co-cultures, goes along with decreased large-scale applicability. Therefore, conditioned media of independent cell cultures could be used for testing the interplay between cell types and the reciprocal effects. Alternatively, specific compounds that activate NASH promoting-pathways can be added to the cell culture media. Nonetheless, direct cell–cell contacts and thus co-culture models will be necessary to study novel, unknown processes related to steatosis-preceding inflammation and defining determinants of MAFLD progression [114].

## 3. Dual-Etiology in MAFLD: Fatty Liver with Multiple Faces

Unlike the terminology of NAFLD, where the disease could only be diagnosed in the absence of other well-defined liver pathologies, MAFLD embraces the existence of dual etiologies. This implicates that MAFLD can coexist with concurring pathologies such as drug-induced liver injury (DILI), viral hepatitis and moderate alcohol consumption, which has been observed in clinical practice as well [115,116,117,118]. Hence, a patient suffering from any other liver disease and who also meets the criteria for diagnosis of MAFLD, should be defined as having dual (or more) etiology fatty liver disease [119].

### 3.1. Drug Intake

Certain pharmaceuticals are known to induce liver steatosis and inflammation [120]. However, some of these compounds might be of vital importance or may belong to a ‘no switch’ drug class, making their continuous intake necessary for the well-being of the patient. Sodium valproate (VPA), indicated for the treatment of epilepsy, can serve as an example. Using stem cell-derived hepatocyte-like cells and HepaRG cultures, it has been shown that VPA-induced steatosis can be evaluated using in vitro cell models [121,122]. Furthermore, as the natural history of the donor can influence metabolic pathways that are relevant for the disease under study, thorough drug intake screening of donors of primary human hepatocytes is essential when using these cells for modeling MAFLD subtypes in vitro. Moreover, not only steatogenic drugs such as VPA may influence data obtained from cell cultures. Indeed, fourteen day-repeated exposure of primary human hepatocyte spheroids to acetaminophen resulted in hepatotoxicity and decreased cell viability [123]. Furthermore, exposure of cocultures of HepaRG cells and primary human hepatic stellate cells to acetaminophen induced hepatocyte damage along with stellate cell activation. Similarly, in the same study it was found that repeated exposure of these spheroids for fourteen days to methotrexate, a drug indicated for neoplastic diseases and severe rheumatoid arthritis or psoriasis, leads to stellate cell activation [124]. These observations suggest that results obtained using cultures from donors with a long-term history of acetaminophen or methotrexate intake should be interpreted with caution.

### 3.2. Ethanol Consumption

Conflicting data exists regarding the effects of ethanol consumption in patients suffering from MAFLD. A prospective cohort study including 4568 NAFLD patients concluded that modest ethanol consumption (i.e., 0.5 to 1.5 standard drinks per day) decreases all-cause mortality compared to ethanol abstinence. In contrast, drinking more than 1.5 standard drinks of ethanol per day, rapidly increases all-cause mortality [125]. These findings are consistent with the fact that moderate ethanol intake reduces the risk for several cardiovascular outcomes [126] and that the beneficial or detrimental effects of ethanol on different health aspects follow a ‘J-shaped’ curve [125]. Prohibiting ethanol consumption in clinical studies is far from practical, often does not reflect the daily situation and would narrow the definition of MAFLD [7,127]. Therefore, pharmacological effects of novel anti-NASH compounds could be evaluated in vitro with different alcohol concentrations to gain insights in situations that reflect daily life for many patients. Ethanol induces transcriptional activity of SREBP-1c and carbohydrate-responsive element-binding protein thereby promoting lipogenesis [128]. SREBP-1c levels, however, decrease in patients with NASH progression [129]. Concomitant exposure of FaO hepatoma cells to ethanol and free fatty acids reduces lipid droplet size compared to single treatments [130], which correlates with more advanced MAFLD histology [131]. Coexisting alcoholic- and non-alcoholic fatty liver disease might therefore be an important but underrecognized disease. Evaluation of compounds using in vitro systems, in which both the effects of ethanol and metabolic factors are modeled, can facilitate the development of drugs targeting mixed pathologies.

### 3.3. Viral Hepatitis

In clinical practice, MAFLD and chronic hepatitis B (CHB) are increasingly observed together, with a significant impact on clinical outcome. In patients exhibiting dual NASH/CHB etiology, disease progression is rapid, along with accelerated manifestation of advanced fibrosis, liver-related outcomes and death [118]. Similarly, fibrosis progression and development of HCC are accelerated in patients suffering from both NAFLD and chronic hepatitis C virus (HCV) infection [116]. In addition, hepatic steatosis due to metabolic factors, significantly hampers interferon-based treatment of HCV, suggesting the need for a holistic approach [132,133]. Direct-acting antiviral (DAA) treatment of HCV is seemingly not hampered by metabolic steatosis [134]. However, in patients with genotype 1 chronic hepatitis C infection, who suffer predominantly from metabolic steatosis, an increase compared to baseline in the liver steatosis marker controlled attenuation parameter (CAP) has been reported [135]. This adverse response to DAA treatment requires further investigation in the context of dual etiology fatty liver [136]. The mechanisms of interplay between MAFLD and viral hepatitis are still unclear and need further elucidation, for which human-based in vitro methodologies may serve as a practical testing platform [137]. Whereas established techniques to include HCV in vitro exist [138], modeling CHB appears to be more difficult. A major hurdle to include CHB in an in vitro model is the rapid loss of sodium/bile acid cotransporter (NTCP), which serves as the entry receptor for the virus. Although primary hepatocytes express *NTCP*, infection susceptibility decreases shortly after plating, due to dedifferentiation and high variability between donors that exists due to genetic susceptibility to infection. This can somewhat be tackled with specialized cell-culture systems, e.g., by using micropatterned cocultures with stromal cells. To address variation in susceptibility to infection, iPSC-derived hepatocyte-like cells were successfully applied [139]. Furthermore, HepaRG is the only hepatoma cell line to date that expresses *NTCP*, albeit at low levels in their undifferentiated state. HepaRG therefore requires lengthy differentiation, consisting of two weeks of maintaining the undifferentiated cells in culture and two to four weeks of induction using corticoids and dimethylsulfoxide, into a hepatocyte-like and biliary-like cell mixture before *NTCP* is significantly upregulated. The liver cell lines HepG2 and Huh7 completely lack *NTCP* expression, which can be addressed by inducing constitutive expression of *NTCP* via reprogramming [140].

## 4. Discussion

**Figure 3:**
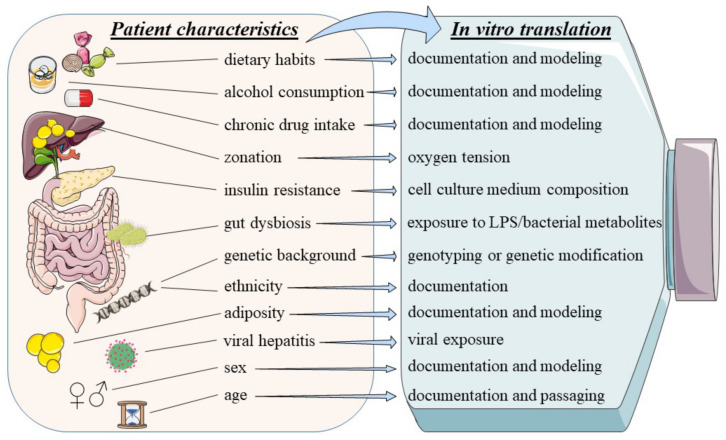
Translation of MAFLD patient characteristics to an in vitro environment. Multiple factors contribute to the phenotypical presentation of MAFLD. Cells used for setting up in vitro MAFLD disease models may inherently exhibit disease modifiers that require proper documentation of their origin in the first instance. Well-established culture conditions including non-physiological cell culture medium composition and oxygen tension may also influence specific pathogenic mechanisms. [Abbreviations: LPS: lipopolysaccharide].

Adjustment of the NAFLD terminology has been implemented to advance anti-NAFLD drug development and mainly to redesign clinical studies. It is, however, of utmost importance to align this novel mindset to basic mechanistic and translational research in order not to generalize anti-MAFLD drug development in the early preclinical stages. A key point in translational in vitro MAFLD research is proper documentation of the used cells in culture including data on sex, age, ethnicity and lifestyle of the donor, since taking into account this variability is essential for result interpretation (Figure 3).

Anti-NASH therapy will possibly consist of a combinatory program in which patients are actively encouraged to undertake physical activity along with pharmacological treatments to stimulate disease remission, which in turn will probably also consist of combinatorial therapy given the heterogeneity of the disease. However, particular caveats in early anti-NASH drug development can be noticed due to patient heterogeneity related to (epi)genetics, differences in diet, ethnicity, ethanol consumption, gut microbiota, sex and drug intake. Although these disease-modifiers can be deduced from translational research and patient studies, each feature will likely not contribute to disease onset and/or development in an individual manner. Moreover, one might question how the general terminology of MAFLD reflects groups of patients with seemingly different etiologies. MAFLD holds broad diagnostic criteria and might be too sensitive across the general population [141,142]. From a translational point of view, early drug development would perhaps benefit from more disease-specific terminology such as “obesity associated fatty liver disease (OFLD)” or “diabetes associated fatty liver disease (DFLD)”, as suggested by Poniachik et al. [141].

On the other hand, since fibrosis is the most important determinant for disease-specific mortality in NAFLD [143], one might question the importance of detailed modeling of MAFLD subtypes in order to develop effective therapeutics. Instead, drug development could be focusing solely on the fibrosis aspect of MAFLD. Nevertheless, since previously developed anti-NASH drugs have been targeting fibrosis/hepatic stellate cells as well, e.g., simtuzumab [144] and elafibranor [145], without satisfying results, it still seems the way to go. Adopting the new MAFLD terminology seems to be more practical for identification of high-risk patients, as it was found to allow better identification of patients with significant hepatic fibrosis than when the NAFLD terminology was applied [146].

Considering the multi-faced pathogenesis of MAFLD, inter-species differences are likely to bias early basic and translational studies [2]. The use of primary human hepatocytes in translational MAFLD research, therefore, seems an attractive strategy [147]. Nonetheless, primary human hepatocytes are not only scarce, but often also derived from donors with an unprecedented history related to, e.g., drug, diet and alcohol intake. The most important advantages and disadvantages of hepatic cell systems to consider for modeling MAFLD in vitro are shown in Table 3.

Despite the recent advancements in the expansion of primary human hepatocytes in vitro [152], the contributions of diet, physical activity and ethanol intake on the NASH pathogenesis seem to largely impede their application in MAFLD research. Moreover, one might question whether primary human hepatocytes should still be considered the gold standard for in vitro experimentation when studying MAFLD if their origin is not well documented nor made available. Human stem cell-derived hepatocyte-like cells and induced hepatocytes might therefore represent valid alternatives. Although fully mature induced hepatocytes are difficult to obtain for clinical applications due to safety issues, their implementation in basic and translational pharmacological in vitro research seems eminent [153]. The use of iPSC- or other stem cell-derived hepatic cells obtained from donors with different MAFLD etiologies might enable the construction of tailored in vitro models during early drug development, anticipating patient stratification in clinical trials, and hence reduce failures during late-phase clinical studies.

## Figures and Tables

**Table 1:** Human-based in vitro models of NAFLD. [Abbreviations: hSKP-HPC: human skin-derived precursor hepatocyte-like cell, IL-1β: interleukin 1 beta, iPSC: induced pluripotent stem cell, LPS: lipopolysaccharide, NAFLD: non-alcoholic fatty liver disease, PPAR: peroxisome proliferator-activated receptor alpha, TGF-β: transforming growth factor beta, TNF-α: tumor necrosis factor alpha].

Cell Type(s)	In Vitro Disease Trigger(s)	Etiology	Pharmacological Intervention	Ref.
In vitro models using primary cells				
Primary hepatocytes	Oleic acid and palmitic acid	Diet	Pioglitazone and metformin	[14]
	Glucose, insulin, free fatty acids, TNF-α, IL-1β and TGF-β	Diet Inflammation	PPAR-agonists	[15]
Primary hepatocytes, stellate cells and macrophages	Glucose, insulin, free fatty acids	Diet Insulin resistance	Obeticholic acid	[16]
	Fructose	Diet	No pharmacological intervention	[17]
Stem cell-based in vitro models				
hSKP-HPC	Glucose, insulin, free fatty acids, TNF-α, IL-1β and TGF-β	Diet Inflammation	Elafibranor	[18]
iPSC-derived hepatocyte-like cells	Lactate, pyruvate and octanoate	Mitochondrial dysfunction	No pharmacological intervention	[19]
	Oleic acid	Diet Genetics	No pharmacological intervention	[20]
	Donor NAFLD background	Genetics	No pharmacological intervention	[21]
	Donor NAFLD background	Genetics	No pharmacological intervention	[22]
iPSC-derived hepatocyte-like, stellate cell-like and Kupffer cell-like cells	Oleic acid and LPS	Gut dysbiosis	Obeticholic acid	[23]
In vitro models using cell lines				
HepaRG	Oleic acid	Diet	PPAR-agonists	[24]
HepaRG and HepG2	Glucose, insulin, free fatty acids, TNF-α, IL-1β and TGF-β	Diet Inflammation	PPAR-agonists	[15]
Huh7 and LX-2 cells	Oleic acid and palmitic acid	Diet-induced fibrosis	No pharmacological intervention	[25]

**Table 2:** Strengths and limitations of cell culture systems for hepatic in vitro modelling.

System	Strengths	Limitations
Monolayer cultures [26,27]	- Low cost - Relatively simple handling - Convenient for analysis - Characterized and benchmarked - Suitable for high-throughput purposes	- Abnormal cell morphology - Loss of polarization - Rapid dedifferentiation, not suitable for long-term studies - Hampered cell–cell contacts - No representation of interorgan crosstalk
Sandwich cultures [26,27,28]	- Longer lifespan and preservation of metabolic activity - Decreased cellular flattening - Improved cell–cell contacts	- Renewal of overlay is required every couple of days to decelerate dedifferentiation - Vulnerable to batch-to-batch variation of extracellular matrix substrates - Limited exchange of nutrients and compounds due to extracellular matrix overlay - Less suitable for high-throughput purposes - No representation of interorgan crosstalk
Micropatterned co-cultures [26,29]	- Phenotypic stability over several weeks - Controlled degree of homo- and heterotypic cell–cell contacts	- Higher cost - Murine supporting cell line (3T3-J2) often used is a concern for physiological relevance - Interference of supporting cells with read-outs - No representation of interorgan crosstalk
Spheroids [28,30]	- Cell–cell interactions - Preservation of functional and metabolic activity over several weeks - Do not require scaffolds - Suitable for high-throughput purposes	- Size heterogeneity (depending on procedure) - No representation of interorgan crosstalk
Organoids [31]	- Cell–cell interactions - Preservation of functional and metabolic activity over several weeks - Recapitulate developmental phases of the liver	- Higher cost - Require scaffolds - High variability due to lack of standardized protocols - Limited options for genetic modification - Limited representation of interorgan crosstalk
Microfluidic devices [26,27]	- In vitro physiological liver environment - Cell–cell interactions - Perfusion, shear stress - Zonation possible by managing flow rate of medium - Inclusion of interorgan crosstalk	- High cost - High complexity - Requires specialized equipment - Less suitable for high-throughput purposes
Bioprinting [26,32]	- In vitro physiological liver environment - Cell–cell interactions - Vascularization	- High cost - Complex experimental setup - Limitations concerning cell viability and structural integrity depending on printing method, requires large amounts of cells - Less suitable for high-throughput purposes

**Table 3:** Strengths and limitations of hepatic cell sources for modeling MAFLD.

	Strengths for MAFLD Modeling	Limitations for MAFLD Modeling
Primary human hepatocytes [148,149,150,151]	- excellent drug-metabolizing capacity - preserved lipid and lipoprotein metabolism	- scarcity of donor material - uncertain dietary history - uncertain alcohol intake history - uncertain drug intake history - limited lifespan - uncertain presence of pathogen- and damage-associated molecular patterns - often already unhealthy donors
Hepatoma-derived cell lines [149,150,151]	- easy genetic adaptations - long-term culture possible	- lack of population diversity - sex-specific - went through multiple passages - poor drug-metabolizing capacity - altered lipid and lipoprotein metabolism
Stem cell-derived models [149,150,151]	- unlimited source of cells - no ethical concerns - studying epigenetics - easy genetic adaptations - long-term culture possible - can obtain comparable lipid and lipoprotein metabolism to primary cells - population diversity—can be obtained from different donors	- limited drug-metabolizing capacity - long differentiation protocols - cryopreservation difficulties

## Data Availability

Not applicable.
